# Dynamic Activity of Histone H3-Specific Chaperone Complexes in Oncogenesis

**DOI:** 10.3389/fonc.2021.806974

**Published:** 2022-01-11

**Authors:** Ting Wen, Qiao Yi Chen

**Affiliations:** Department of Cell Biology and Genetics, School of Basic Medical Sciences, Xi’an Jiaotong University, Xi’an, China

**Keywords:** histone, histone variants, H3.1, H3.3, chaperone, cancer

## Abstract

Canonical histone H3.1 and variant H3.3 deposit at different sites of the chromatin *via* distinct histone chaperones. Histone H3.1 relies on chaperone CAF-1 to mediate replication-dependent nucleosome assembly during S-phase, while H3.3 variant is regulated and incorporated into the chromatin in a replication-independent manner through HIRA and DAXX/ATRX. Current literature suggests that dysregulated expression of histone chaperones may be implicated in tumor progression. Notably, ectopic expression of CAF-1 can promote a switch between canonical H3.1 and H3 variants in the chromatin, impair the chromatic state, lead to chromosome instability, and impact gene transcription, potentially contributing to carcinogenesis. This review focuses on the chaperone proteins of H3.1 and H3.3, including structure, regulation, as well as their oncogenic and tumor suppressive functions in tumorigenesis.

## Introduction

In eukaryotes, histone proteins wrap around the DNA to form nucleosomes, which are the building blocks of chromatin. Each nucleosome core particle is composed of around 147bp of DNA and an octamer of histones that is formed of two H3/H4 dimers and two H2A/H2B dimers. Histone H1 is a linker histone that connects adjacent nucleosomes (1). Aside from the canonical histones, H2A, H2B, H3, and H4, histone variants play a pivotal role in regulating chromatin dynamics and the accessibility of the underlying DNA in a locus-specific manner (2). In contrast to the canonical histone, non-canonical histone variant genes locate outside the histone gene clusters, contain introns and their mRNAs have poly(A) tails, which increase the histone diversity. In addition, canonical histones are expressed and incorporated into the chromatin during DNA replication in the S phase (3–6), whereas the assembly of histone variants is replication-independent and spans all phases of the cell cycle (2, 7). Thus non-canonical histone variants may play important roles in other DNA-dependent processes outside the S phase, such as transcription initiation and elongation (8).

**Figure 1:**
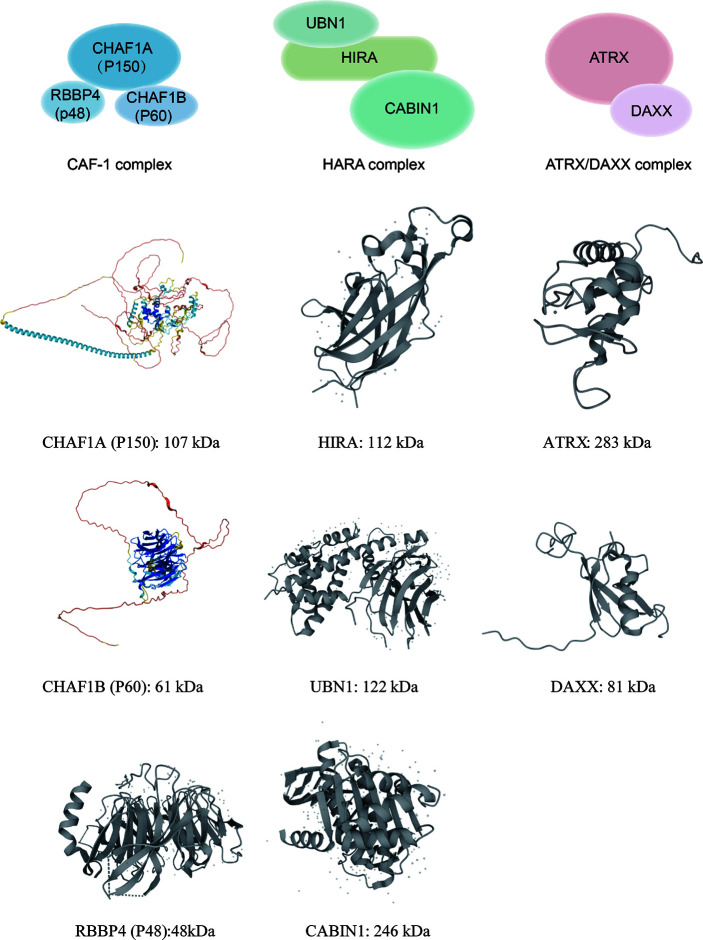
This graph illustrates annotated domains of histone chaperone complexes, along with individual 3D structures of chaperone subunits (3D images from Uniprot 2021).

In human somatic cells, seven variants of H3 (H3.1, H3.2, H3.3, CENP-A, H3.1T, H3.X, and H3.Y) have been identified (6). Canonical histone variants H3.1 and H3.2 are termed replication-coupled histones because they are incorporated during DNA replication. In addition, H3.2 differs from H3.1 by only one amino acid. Non-canonical histone variant H3.3 is encoded by H3F3A and H3F3B in humans, and it differs from H3.1 by only five amino acids (6). H3.3 dysregulation is implicated in a variety of biological processes: embryonic stem cell differentiation, epigenetic reprogramming, neuron plasticity, centromere maintenance, and DNA damage response (9). To ensure the temporal and spatial correctness of histone functions, histone needs the chaperones to bind histones directly after their synthesis. By definition, histone chaperones are a group of proteins that neutralize the positive charge of histones to prevent non-specific interactions between histones and DNA (10, 11). They are involved in the storage, exchange, and deposition of histones on DNA for assembly (12). Different histone chaperones mediate the deposition of canonical histones and histone variants. Canonical histone H3.1 relies on chaperone anti-silencing factor 1A (CAF-1) to incorporate into chromatin in a replication-dependent manner. H3.3 variant uses specific chaperones: HIRA, DAXX/ATRX complex, and DEK to incorporate into the chromatin in a replication-independent manner (13). Chaperones have been shown to prefer distinct sites for H3.3 assembly. For example, H3.3 requires HIRA to promote its deposition at transient nucleosome-free regions, while DAXX/ATRX is necessary for H3.3 enrichment at heterochromatin (13, 14). DEK maintains chromatin integrity by controlling H3.3 deposition into specific genomic regions (6, 15, 16). Increasing numbers of researchers have demonstrated that histone chaperones are frequently mutated in tumors, indicating that they play a key role in tumorigenesis. Here, we focus on the potential functional roles of histone chaperone proteins CAF-1, HIRA, and ATRX/DAXX in carcinogenesis (**Figure 1**).

## Histone H3.1 Chaperone: CAF-1 Complex

As an evolutionarily conserved H3/H4 histone chaperone, CAF-1 complex was first identified in DNA replication experiments (17). In humans, CAF-1 consists of three subunits, P150(CHAF1A), P60(CHAF1B), and P48(RBBP4), that were named based on their apparent molecular weight following gel electrophoresis (17). The CHAF1A subunit contains a Winged-Helix Domain (WHD) that binds DNA in a sequence-independent manner. CHAF1A can interact with Proliferating Cell Nuclear Antigen (PCNA) to target the CAF-1 complex at the replication fork. The CHAF1B subunit can deliver H3/H4 by directly interacting with anti-silencing function 1 (ASF1). The P48 subunit contributes to the interaction of histone-modifying enzymes and their substrates. P48 subunit binds independently to fragments of H3/H4 using different interaction surfaces. Overall, the function of CAF-1 complex is to deliver newly synthesized H3/H4 dimers to the replication fork during S phase of cell cycle (18) and participate in DNA damage repair.

## CAF-1 and Cancer

The CHAF1B subunit is overexpressed in a variety of tumors, including high-grade glioma, melanomas, prostatic, renal, cervical carcinomas, endometrial tumors, hepatocellular, squamous cell carcinoma, salivary gland tumors, leukemia, and breast cancer (19–29) (**Table 1**). Moreover, CHAF1B is a major factor for driving metastasis in many different human tumors, as increased protein levels can be used to accurately predict whether or not these tumors will metastasize (18). In hepatocellular carcinoma (HCC), knockdown of the CHAF1B gene reduced the migration and invasion ability of HCC cells, suggesting that the CAF-1 may function as an oncogene (22). In breast cancer, CAF-1 has been shown to be a useful proliferation marker (24). However, in another study, the downregulation of CAF-1 was found to promote the progression of breast cancer (30). Gomes et al. revealed that extracellular regulated protein kinases (ERK) -dependent transfer signal promotes a switch in H3 variants incorporated into chromatin by down-regulating histone chaperones CAF-1 (31). In carcinoma cell lines, the ERK2 signaling reduces the levels of H3.1/H3.2 by suppressing CHAF1B transcription, thus creating the “space” for gap-filling with H3.3, leading to a HIRA-dependent H3.3 enrichment at the promoter of EMT, resulting in tumor progression and metastasis formation. There are few studies focused on CHAF1A, although some have reported its reduction in squamous cell carcinoma (26) and breast cancer (30), indicating potential and anti-cancer effects. Overall these studies indicate that histone chaperones may be valuable therapeutic targets for aggressive tumors.

**Table 1:** Expression of CAF-1 in tumor.

Cell Type/cancer type	Cancer Expression Level	Function	References
High-grade glioma	Increased (CAF-1/p60)	Cancer Promoting	(19, 20)
Melanomas	Increased (CAF-1/p60)	Cancer Promoting	(21)
Prostatic cancer	Increased (CAF-1/p60)	Cancer Promoting	(25)
Renal carcinomas	Increased (CAF-1/p60)	Cancer Promoting	(23)
Cervical cancer	Increased (CAF-1/p60)	Cancer Promoting	(23)
Cervical cancer	Increased (CAF-1/p150)	Cancer Promoting	(29)
Endometrial tumors	Increased (CAF-1/p60)	Cancer Promoting	(23)
Hepatocellular carcinoma	Increased (CAF-1/p60)	Cancer Promoting	(22)
Squamous cell carcinoma	Increased (CAF-1/p60)	Cancer Promoting	(26)
Salivary gland tumors	Increased (CAF-1/p60)	Cancer Promoting	(27)
Breast cancer	Increased (CAF-1/p60)	Cancer Promoting	(24)
Leukemia	Increased (CAF-1/p60)	Cancer Promoting	(28)
Squamous cell carcinoma	Decreased (CAF-1/p150)	Cancer Promoting	(26)
Breast cancer	Decreased (CAF-1)	Cancer Suppressing	(30)

## Histone H3.3 Chaperone: HIRA Complex

The histone cell cycle regulator (HIRA) is an evolutionarily conserved H3/H4 histone chaperone (32, 33). Human HIRA was originally identified in DiGeorge syndrome patients, who commonly have heart and brain abnormalities (34), and later described as a histone chaperone (35). The HIRA complex is composed of HIRA, Ubinuclein-1 (UBN1), and calcineurin-binding protein 1 (CABIN1), which coordinate with ASF1 to bind and deposit H3.3/H4 into the chromatin in a DNA replication-independent manner (36, 37). The HIRA subunit can enhance the binding affinity of UBN1 towards H3.3. The UBN1 subunit is mainly responsible for specific recognition and direct binding of H3.3 (38, 39). In addition to these core partners, HIRA can also directly interact with ASF1b or ASF1a and transfer H3.3/H4 dimers to HIRA complexes (40).

The HIRA complex deposits H3.3 mainly at euchromatin regions such as promoters, enhancers, actively transcribed gene bodies, gene regulatory regions, developmentally regulated genes, and areas of DNA and chromatin damage and repair (37, 41, 42). The HIRA complex interacts with the single-stranded DNA (ssDNA)-binding protein replication factor A (RPA) to deposit newly synthesized H3.3 at gene transcription regulatory elements (42). It has been reported that HIRA can promote transcription recovery after DNA damage as well as maintain global nucleosomal architecture and genomic integrity (32, 43, 44). Furthermore, HIRA binds to naked DNA *in vitro* and non-nucleosomal regions *in vivo*, suggesting that deposition of H3.3-gap filling is HIRA-dependent (45). Interestingly, some has suggested that HIRA-mediated H3.3 deposition may be a mechanism to maintain genomic stability when chaperone protein CAF-1 mediated H3.1 deposition is impaired during S-phase (6). Various lines of research suggest that HIRA is involved in a range of processes including embryonic development (46, 47), angiogenesis (48, 49), cellular senescence (50, 51), and early neural development (52).

## HIRA and Cancer

HIRA is involved in cellular senescence and is closely related to cancer carcinogenesis (**Table 2**). Cellular senescence is an irreversible proliferation arrest triggered by short chromosome telomeres, activated oncogenes, and cellular stress. Furthermore, cellular senescence is a known tumor suppressor mechanism. Hall et al. demonstrated that HIRA can interact with Cyclin-CDK2, whose expression blocks S-phase progression and promotes cellular senescence (54). HIRA can interact with ASF1a to form facultative heterochromatin called senescence-associated heterochromatin foci (SAHF), thereby inhibiting cell proliferation and causing cell senescence (50, 51). Another study found that the HIRA mRNA overexpression in chronic myeloid leukemia (CML). Majumder et al. demonstrated that the downregulation of HIRA could induce the differentiation of CML cells and inhibit their proliferation (53). Similarly, in a metastasis-induced breast cancer model, a pronounced upregulation of HIRA and a decrease of CAF-1 can be observed (30). As a histone chaperone that mediates H3.3 gap-filling, knockdown of HIRA suppresses the migration and invasion of human breast cancer cell lines LM2 (30). These studies suggest that HIRA may serve as a potentially effective therapeutic target for metastatic cancer.

**Table 2:** Expression of HIRA in tumor.

Cell Type/cancer type	Cancer Expression Level	Function	References
Chronic myeloid leukemia cells	Increased	Cancer Promoting	(53)
Breast cancer	Increased	Cancer Promoting	(30)

## Histone H3.3 Chaperone: ATRX/DAXX Complex

In addition to HIRA, the ATRX/DAXX complex is another H3.3 chaperone protein. Alpha-thalassemia X-linked intellectual disability (ATRX) and death domain-associated (DAXX) proteins localize to promyelocytic leukemia nuclear bodies (PML­NBs), which are multipurpose subnuclear domains implicated in transcriptional activation, DNA replication, apoptosis, and viral infection (55–57). The function of the ATRX/DAXX complex as an H3.3-specific deposition complex was identified through the purification of histone variant chaperone complexes (58–60). The ATRX gene was first identified in patients with the ATRX syndrome (61). ATRX encodes a 2,492 amino acid protein with a molecular weight of 282,586 Da (62). The ATRX protein is a chromatin remodeling factor initially described as a putative helicase protein due to sequence homology with the DNA repair and recombination Rad54 protein (63, 64). The ATRX protein contains two highly conserved domains, an ADD (ATRX-DNMT3-DNMT3L) domain in the N-terminal and an ATPase/helicase domain in C-terminal (65–68). The ADD domain can recognize H3K9me3­containing nucleosomes in the absence of H3K4 methylation (69), so ATRX itself is an efficient reader of the H3K9me3 histone mark *via* this domain (66, 70–75). The ATPase/helicase domain belongs to the SWI/SNF2 (SWItch/Sucrose Non Fermentable) family of chromatin remodeling proteins (76). It also contains a plant homeodomain (PHD) zinc finger domain, which is most similar to the DNA methyltransferase 3 family of proteins (77). When ATRX binds to nucleosomes or DNA, the ATPase chromatin remodeling activity of the ATPase/helicase domain can be activated (77, 78).

DAXX was originally identified as a fatty acid synthase (FAS) binding protein that induced apoptosis *via* Jun N-terminal kinase (JNK) pathway (79) and further work identified it as a chaperone of histone variant H3.3 (58). DAXX preferentially binds to promoter regions and regulates H3.3 loading of immediate early genes after neuronal stimulation (14, 80). DAXX is a transcription repressor that interacts with histone deacetylases (HDAC) and DNA methyltransferases (81). DAXX has four domains: the DAXX helical bundle (DHB); DAXX histone-binding domain (HBD); DAXX acidic domain and SUMO-interacting motifs (SIMs) (82). The DHB domain contains a defined binding surface for a number of DAXX-interacting proteins such as ATRX, Ras-association domain family 1 isoform C (RASSF1C), p53, and mouse double minutes 2 homolog (MDM2) (83). The HBD domain binds the H3.3/H4 dimer for H3.3-specific recognition (84). Crystal structure analysis revealed that DAXX distinguishes H3.3 through direct interaction with the variant-specific residues (87-90) in the core histone fold of H3.3 (6, 60). The DAXX acidic domain appears to increase the binding affinity to the H3.3/H4 dimer. DAXX has two SIMs, located at the N- and C-terminus respectively. The four domains are closely related to the regulation of DAXX transcription.

## The Interaction of DAXX and ATRX

**Figure 2:**
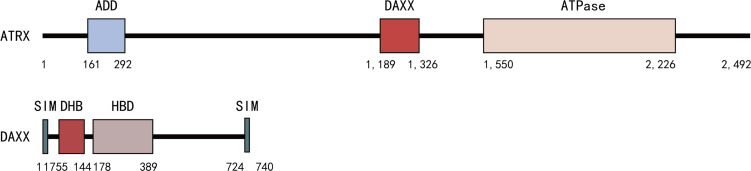
DAXX binds to the linker region of ATRX (residues between 1,189 and 1,326) located between the ADD and ATPase domains through its N-terminal DHB domains.

The ATRX/DAXX complex is an ATP-dependent chromatin remodeling complex, with ATRX being the core ATPase subunit and DAXX being the targeting subunit (77). DAXX binds to the linker region of ATRX (residues between 1,189 and 1,326) located between the ADD and ATPase domains through its N-terminal DHB domain (77, 85) (**Figure 2**). ATRX binds H3K9me3 *via* its ADD domain and heterochromatin protein 1 (HP1) *via* the PxVxL motif, thereby recruiting DAXX to heterochromatin regions (86). Besides, the binding affinity of DAXX/ATRX is stronger than DAXX and RASSF1C, p53, or MDM2, mainly due to additional electrostatic interactions between positively charged residues in 4HB and negatively charged residues in ATRX (81, 83, 85, 87).

## ATRX and Cancer

There are two known Telomere Maintenance Mechanisms (TMMs): telomerase-mediated telomere maintenance and telomerase-independent telomere maintenance mechanism termed alternative lengthening of telomeres (ALT) (88, 89). The ALT pathway in cancer was first identified in 1997 (90). Unlike the HIRA complex, the ATRX/DAXX complex facilitates H3.3 deposition at heterochromatin, such as telomeres (7, 60). As a histone chaperone, the loss of ATRX/DAXX will impair H3.3 loading at telomeres, leading to ALT and chromosomal instability (CIN) (91–95). Through telomere-specific fluorescence *in situ* hybridization (FISH), Heaphy et al. revealed that all pancreatic neuroendocrine tumors (PanNETs) samples with ATRX or DAXX gene mutations displayed large, ultrabright telomere FISH signals. This is a universal feature of ALT (92), suggesting that the mutations of ATRX and DAXX are highly related to the ALT. The ALT was associated with DAXX or ATRX mutations in adult adrenocortical tumorigenesis, but exclusively with ATRX mutations in pediatric cases (96, 97). ATRX represses ALT and is required to maintain genomic stability (98). However, Liu et al. demonstrated that there is no significant association between ATRX mutation/loss of expression and ALT in adult diffuse astrocytic tumors (99). In addition, another study showed that in mouse embryonic stem cells, although ATRX loss causes extensive genomic instability, it does not on its own cause ALT or cancer (94, 100). Consistent with this, a study led by Schwartzentruber et al. demonstrated that the simultaneous presence of *ATRX/H3F3A/TP53* mutations was highly related to ALT (101). The gene *H3F3A*, which encodes histone variant H3.3, was recurrently mutated in the pediatric glioblastoma multiforme and led to the critical amino acid mutations at the histone tail (K27M, G34R/V). Of note, mutations in ATRX overlapped significantly with mutations in *H3F3A* and tumor suppressor *TP53* (99, 101, 102), suggesting that there may be collaborative effects among them (76). Intriguingly, H3.3-G34R/V mutations co-occur with ATRX mutations (69, 101, 103), whereas H3.3-K27M mutations did not (69). H3.3K27 often undergo important post-translational modifications like methylation, which is commonly associated with transcriptional repression (101). H3.3 mutations induce chromatin remodeling to produce different gene expression profiles for the K27 and G34 mutations. Whole-exome sequencing showed that genes involved in development and differentiation are distinct among H3.3-K27 and H3.3-G34 mutants (101). ATRX loss-of-function will impair H3.3 loading at telomeres and disrupt the heterochromatic state, facilitating ALT (101).

In recent years, with whole-genome sequencings in cancer, ATRX mutations/losses have been detected in a variety of cancers, such as PanNETs (104–106), Glioblastoma multiforme (GBM) (101), neuroblastoma (107), adrenocortical tumor (96, 97), pediatric osteosarcoma (108), angiosarcomas (109), and Gliomas (99). In PanNETs, there is a high ratio of inactivated to missense mutations in DAXX/ATRX, suggesting that they function as tumor suppressor genes. Intriguingly, patients with DAXX/ATRX mutations often show prolonged survival than patients without those mutations (105). In addition, whole-genome sequencing suggests that ATRX is recurrently mutated in osteosarcoma and is also associated with ALT (108). These loss-of-function mutations ranged from point mutations to frameshift insertions/deletions and were mainly localized within the ADD and C-terminal helicase domain (69, 101, 105, 110). As discussed above, while ATRX loss-of-function is found in various tumors, overexpression of ATRX has been reported in colorectal cancer cell lines (64, 111) (**Table 3**). For instance, Athwal et al. demonstrated that ATRX is overexpressed in colon cancer SW480 cells (111).

**Table 3:** Expression and mechanism of ATRX in tumor.

Cell Type/cancer type	Cancer Expression Level	Functional consequences	References
Uterine leiomyomas UL subtype	Decreased	Activation the ALT pathway	(112)
LAPC4 prostate cancer cells	Decreased	Activation the ALT pathway	(113)
Glioblastoma multiforme (GBM)	Decreased	Activation the ALT pathway	(101)
Angiosarcomas	Decreased	Activation the ALT pathway	(109, 114)
Gastrointestinal stromal tumors	Decreased	Activation the ALT pathway	(115)
PanNETs	Decreased	Activation the ALT pathway	(104–106, 116)
Melanoma	Decreased	Activation the ALT pathway	(110)
GBM	Decreased	Activation the ALT pathway	(101)
Pediatric osteosarcoma	Decreased	Activation the ALT pathway	(108)
Neuroblastoma	Decreased	Activation the ALT pathway	(107)
Adrenocortical carcinoma	Decreased	Activation the ALT pathway	(96, 97)
Adult diffuse astrocytic tumors	Decreased	Related to IDH1/2 and TP53 mutations	(99)
Colorectal cancer cell lines	Increased	Related to overexpression of CENP-A	(111)

## DAXX and Cancer

DAXX mutations more frequently occur in the regions that interact with ATRX and the H3.3/H4 dimer, suggesting that the loss of H3.3 chaperone function of DAXX may lead to abnormal chromatin structures, epigenetic dysregulation, and chromosome instability (82). DAXX mutations are relatively rare compared to *H3F3A* and ATRX (101). For example, in neuroblastomas, ATRX loss-of-function mutations play a role in ALT which is related to worse prognosis (117). However, DAXX mutations were not detected in neuroblastomas. DAXX gene expression is not significantly changed in ALT-positive neuroblastomas (117). In addition, mutations in ATRX and DAXX were mutually exclusive (92, 101, 105), confirming that they function together in the same pathway. The expression of DAXX is often dysregulated in tumor cells (**Table 4**). For example, DAXX is overexpressed in many types of cancer such as prostate cancer (118, 120, 130), ovarian cancer (121, 122), gastric cancer (123), and gliomas (14). DAXX is downregulated in advanced gastric cancer (125) and human colon adenocarcinoma cells (126, 127), lung cancer (128), and PanNETs (129). One study suggests that DAXX binds to anaphase promoting complex (APC) coactivators Cdc20 and Cdh1 to inhibit the degradation of APC, thereby promoting chromosome instability during prostate cancer development (118). In addition, Puto et al. demonstrated that in prostate cancer, DAXX binds to DNA methyltransferase (DNMT1) resulting in hypermethylation of the promoter regions of the apoptosis-and autophagy-relevant genes, represses autophagy, and promotes tumorigenicity (119, 120). Overexpression of DAXX promoted ovarian cancer cell proliferation, colony formation, and migration, whereas DAXX depletion by RNA interference had the opposite effects (121). DAXX acts as an oncogene by interacting with PML to protect ovarian cancer cells from DNA damage (121). A subsequent study showed that DAXX promotes ovarian cancer ascites cell proliferation by activating the ERK pathway and directly binding to CCAAT enhancer binding protein-beta (CEBP-β) (122). Benitez et al. proposed a model that DAXX removes H3.3 from the chromatin by competing for chromatin binding to promote oncogene transcription in PTEN-deficient PTEN-null cells (14). In oral squamous cell carcinoma (OSCC) human samples and cell lines, DAXX expression was frequently upregulated. A study showed that DAXX silencing in OSCC cells suppresses cyclin D1 expression *via* the DAXX-TCF4 (transcription factor 4) interaction, thereby reducing tumor growth (124). In gastric cancer, nuclear/cytoplasmic ratio (NCR) of DAXX expression was found higher in gastric cancer tissues than adjacent normal tissues (123). However, the expression of DAXX was decreased in advanced gastric cancer samples. The upregulation of DAXX in gastric cancer cells inhibited proliferation, migration, invasion, and epithelial-mesenchymal transition (EMT). DAXX overexpression inhibited the growth of gastric cancer through downregulating snail family transcriptional repressor 3 (SNAI3), a key inducer of EMT, by recruiting HDAC-1 into the nucleus (125). Similarly, a study lead by Tzeng et al. demonstrated that DAXX suppresses Tcf4 transcriptional activity and induces G1 arrest of colon cancer cells, functions as tumor suppressor (127), and the knockdown of DAXX caused significant cell proliferation and promote metastasis (126). In PanNETs, DAXX/H3.3 complex suppresses target genes including Stanniocalcin 2 (STC2) by promoting H3K9me3 (129), suggesting that DAXX acts as a tumor suppressor. In another study, DAXX functions as a tumor suppressor by inhibiting the HIF-1a/HDAC1/Slug axis in hypoxia-induced lung cancer cells (128). Furthermore, DAXX mutations usually mark the increase of malignancy (119, 129). Similarly, the depletion of H3.3 leads to loss of DAXX, because the HBD domain does not establish a stable conformation without H3.3/H4 binding, revealing that the physiological level of H3.3 is necessary for maintaining the level of DAXX protein (81). Therefore, increased H3.3 levels in cancer cells may augment the oncogenic function of DAXX through increasing protein stability (82).

**Table 4:** Expression and mechanism of DAXX in tumor.

Cell Type/cancer type	Cancer Expression Level	Function	Mechanism	References
Prostate cancer	Increased	Cancer Promoting	Binds to APC coactivators Cdc20 and Cdh1 and inhibits the degradation of APC/binds to (DNMT1) and represses autophagy	(118, 119, 131)
Ovarian cancer cell	Increased	Cancer Promoting	Interacts with PML	(121)
Ovarian cancer ascites cell	Increased	Cancer Promoting	Activate the ERK signaling pathway and bind to CEBP-β	(122)
Gastric cancer	Increased	Cancer Promoting	High NCR of DAXX	(123)
PTEN-null cells	Increased	Cancer Promoting	Remove H3.3 from chromatin	(14)
OSCC	Increased	Cancer Promoting/tumor-promoting	DAXX silencing reduces cyclin D1 expression *via* a D-TCF4 interaction	(124)
Human gastric carcinoma cell line MKN45	Decreased	Cancer Suppressing	Repression of SNAI3 by recruiting HDAC-1 into the nucleus	(125)
Colon cancer cell line Hct116	Decreased	Cancer Suppressing	Suppresses Tcf4/Associated with reduced CD24 expression	(126, 127)
Lung cancer	Decreased	Cancer Suppressing	Suppress the HIF-1a/HDAC1/Slug axis	(128)
PanNETs	Decreased	Cancer Suppressing	Promote H3K9me3	(129)

## Conclusions

Histone chaperones play a critical role in the maintenance of global nucleosomal architecture. Histone chaperone CAF-1 facilitates histone H3.1 deposition in a DNA-synthesis-dependent manner. Mutation of CAF-1 protein reduces the incorporation of H3.1 and H3.2, leading to the increased incorporation of H3.3. Histone variants H3.3 chaperone protein HIRA and ATRX/DAXX mediate DNA-synthesis-independent nucleosome assembly. The chaperone HIRA promotes H3.3 deposition at transient nucleosome-free regions (13). This could be a salvage pathway to maintain chromatin integrity when CAF-1 mediated H3.1 deposition is impaired during DNA replication (6). Although histone variant H3.3 was initially thought to be a marker of transcriptional activation (59), it was later discovered to be deposited into heterochromatic regions *via* ATRX/DAXX, indicating that H3.3 deposition in repetitive regions may contribute to chromatin stability (64). Mutations of histone chaperones DAXX and ATRX reduce the level of histone variant H3.3 (92) and active the ALT pathway in telomerase-negative cancers, suggesting that the incorporation of H3.3 is necessary for telomere maintenance (80, 131–133). In addition, inactivation mutation of ATRX/DAXX can cause a shift towards HIRA-mediated H3.3 deposition (2). Aside from mutations, imbalances between H3.3 and H3.1 or H3.3/H3.1 and its chaperones may also have detrimental effects on genome stability (134, 135). Nye et al. proposed a “chaperone competition” model, in which changes in chaperone expression cause their target histone variants to bind to non-homologous partners, the location of histone variants, thereby potentially promoting tumorigenesis (136). Histone chaperone competition may lead to the incorrect deposition of canonical histones and histone variants, thus results in activating the expression of oncogenes and promoting the occurrence of cancer.

Taken together, both the up-and down-regulation of expression of chaperone proteins can potentially contribute to the occurrence of tumor. The contradictory conclusions discussed above indicate that carcinogenesis is an extremely complex process involving the interaction of multiple proteins and signaling pathways. In addition, the chaperones play an important role in the malignant transformation of tumors and may serve as targets for cancer prevention and treatment. The mechanisms of histone chaperones in tumorigenesis remain to be fully elucidated. Further study should be done in histone chaperones to explore the molecular mechanisms underlying carcinogenesis and chromatin regulation.

## Author Contributions

QC and TW conceived the topic and wrote the manuscript. All authors contributed to the article and approved the submitted version.

## Funding

This study is funded by “Young Talent Support Plan” of Xi’an Jiaotong University No. YX6J010.

## Conflict of Interest

The authors declare that the research was conducted in the absence of any commercial or financial relationships that could be construed as a potential conflict of interest.

## Publisher’s Note

All claims expressed in this article are solely those of the authors and do not necessarily represent those of their affiliated organizations, or those of the publisher, the editors and the reviewers. Any product that may be evaluated in this article, or claim that may be made by its manufacturer, is not guaranteed or endorsed by the publisher.

## Glossary

CAF-1: Anti-silencing factor 1A
HIRA: Histone cell cycle regulator
DAXX: Death domain-associated protein
ATRX: Alpha-thalassemia X-linked intellectual disability
DNA: Deoxyribonucleic acid
mRNA: Messenger RNA
poly(A): Polyadenylic acid
CENP-A: Centromere protein-A
H. sapiens: Homo sapiens
WHD: Winged-Helix Domain
PCNA: Proliferating Cell Nuclear Antigen
ASF1: Anti-silencing function 1
UBN1: Ubinuclein-1
CABIN1: Calcineurin-binding protein 1
ssDNA: Single-stranded DNA
RPA: Replication factor A
CDK2: Cyclin-dependent kinase 2
SAHF: Senescence-associated heterochromatin foci
CML: Chronic myeloid leukemia
PML­NBs: Promyelocytic leukemia nuclear bodies
ADD: ATRX-DNMT3-DNMT3L
SWI/SNF2: SWItch/Sucrose Non Fermentable
PHD: Plant homeodomain
FAS: Fatty acid synthase
JNK: Jun N-terminal kinase
HDAC: Histone deacetylases
DHB: DAXX helical bundle
HBD: DAXX histone-binding domain
SIMs: SUMO-interacting motifs
RASSF1C: Ras-association domain family 1 isoform C
MDM2: Mouse double minutes 2 homolog
HP1: Heterochromatin Protein 1
TMMs: Telomere Maintenance Mechanisms
ALT: Alternative lengthening of telomeres
CIN: Chromosomal instability
FISH: Fluorescence in situ hybridization
PanNETs: Pancreatic neuroendocrine tumors
GBM: Glioblastoma multiforme
ERK: Extracellular regulated protein kinases
CEBP-β: CCAAT enhancer binding protein-beta
OSCC: Oral squamous cell carcinoma
TCF4: Transcription factor 4
NCR: Nuclear/Cytoplasmic Ratio
EMT: Epithelial-mesenchymal transition
SNAI3: Snail family transcriptional repressor 3
STC2: Stanniocalcin 2
